# Characterisation and Stabilisation Mechanisms of Azelaic Acid Nanosuspensions: Insights from a Dual Stabiliser System

**DOI:** 10.3390/pharmaceutics17040439

**Published:** 2025-03-29

**Authors:** Sandra Miočić, Jelena Torić, Marina Juretić, Jelena Đoković, Danijela Randjelović, Snežana Savić, Kristina Ferderber, Biserka-Cetina Čižmek, Jelena Filipović-Grčić

**Affiliations:** 1 R&D, PLIVA Croatia Ltd., Teva Group Member, Prilaz Baruna Filipovića 25, 10000 Zagreb, Croatia; sandra.miocic@pliva.com (S.M.); jelena.toric@pliva.com (J.T.); marina.juretic@pliva.com (M.J.); kristina.ferderber@pliva.com (K.F.); biserka.cetina-cizmek@pliva.com (B.-C.Č.); 2 Faculty of Pharmacy and Biochemistry, University of Zagreb, A. Kovačića 1, 10000 Zagreb, Croatia; 3 Faculty of Pharmacy, University of Belgrade, Vojvode Stepe 450, 11221 Belgrade, Serbia; jelena.djokovic@pharmacy.bg.ac.rs (J.Đ.); snezana.savic@pharmacy.bg.ac.rs (S.S.); 4 Institute of Chemistry, Technology and Metallurgy, National Institute of the Republic of Serbia, University of Belgrade, Njegoševa 12, 11000 Belgrade, Serbia; danijela@nanosys.ihtm.bg.ac.rs

**Keywords:** azelaic acid, chitosan, HPMC, nanosuspension, stabilisation, follicular delivery

## Abstract

**Background/Objectives**: This study investigates the stabilisation mechanisms of azelaic acid nanosuspensions (AZA-NS) prepared by wet media milling (WMM) using hydroxypropyl methylcellulose (HPMC) and chitosan as stabilisers. The aim was to elucidate the physical interactions relevant for stabilisation and to evaluate the effectiveness of a dual stabiliser approach to improve AZA-NS stability. **Methods**: AZA-NS were characterised using Fourier transform infrared spectroscopy (FTIR) to evaluate the chemical interactions, differential scanning calorimetry (DSC) for thermal properties, atomic force microscopy (AFM) to analyse the adsorption of the stabiliser on the AZA surface and X-ray diffraction (XRD) to evaluate the crystallinity. Contact angle and immersion studies were performed to evaluate wettability, and alternative stabilisers were tested for comparison. **Results**: Highly concentrated AZA-NS (up to 20% drug loading) were successfully produced with particle sizes between 326.8 and 541.2 nm, which are in the optimal range for follicular drug delivery. FTIR confirmed stabilisation by adsorption and not by chemical interaction. DSC revealed a melting point depression, indicating a partial disorder of the crystal lattice. AFM imaging showed different adsorption patterns for HPMC and chitosan, suggesting better surface coverage compared to alternative stabilisers. XRD confirmed the retention of the AZA crystalline form after milling. Contact angle and immersion studies showed improved wettability due to the synergistic effects of HPMC and chitosan. Alternative stabilisers showed suboptimal performance, highlighting the superior stabilising potential of the HPMC–chitosan combination. **Conclusions**: This study provides important insights into the dual stabilisation mechanisms and highlights the importance of combining steric and electrostatic stabilisers for the formulation of stable nanosuspensions of medium soluble drugs such as AZA. These results support the development of optimised nanosuspensions with increased stability and improved pharmaceutical applicability.

## 1. Introduction

Nanocrystal technology has proven to be a highly effective approach to improve drug delivery, especially in dermatological applications [1]. By reducing the size of drug particles into the nanometre range, nanocrystals significantly increase the specific surface area, resulting in improved saturation solubility, dissolution rate and thermodynamic activity, leading to overall improved percutaneous absorption and bioavailability of poorly water-soluble drugs [2,3]. When administered topically, nanocrystals have been shown to penetrate the skin barrier primarily via the transepidermal (via the stratum corneum) and transappendageal (follicular) routes, offering advantages such as local drug accumulation and reduced systemic exposure [4,5]. Compared to conventional delivery systems such as liposomes or solid lipid nanoparticles, nanocrystals offer higher drug loading—often up to 100%—as well as longer retention time in the skin and improved formulation stability [6,7]. These properties are particularly beneficial in chronic inflammatory dermatoses such as acne and rosacea, where the prolonged residence time of the active ingredient in the pilosebaceous units and the targeted follicular deposition increase efficacy while reducing irritation [1,8]. Recent studies have also shown that nanocrystals can improve passive diffusion through the stratum corneum by increasing adhesion to the skin surface and forming a supersaturated layer that favours a concentration gradient that promotes absorption [9,10]. In addition, nanocrystals can be formulated into conventional dermal vehicles (e.g., gels, creams, hydrogels) using scalable, solvent-free production methods such as wet milling, making them highly suitable for industrial application [3,11].

Azelaic acid (AZA) is a C9 dicarboxylic acid used to treat acne, rosacea and hyperpigmentation disorders due to its antimicrobial, anti-inflammatory and keratolytic properties [12,13]. However, its topical application is complicated by its low water solubility (~2.4 mg/mL at 20 °C) and pH-dependent ionisation, necessitating high concentrations (15–20%) in commercial formulations, which can lead to skin irritation and dryness [14,15]. Studies have shown that nanonisation of AZA significantly improves its dermal bioavailability and allows dose reduction, thereby attenuating the adverse effects [16,17,18]. Despite promising results, previous approaches have relied primarily on drying techniques to maintain nanocrystal stability and provided only limited insights into the stabilisation mechanisms in aqueous, highly concentrated nanosuspensions.

Although nanosuspensions offer advantages such as high loading, solvent-free formulations and flexible routes of administration, their physical stability remains a major limitation, especially for moderately soluble drugs such as AZA. These systems are prone to aggregation, sedimentation and Ostwald ripening due to the high surface energy and solubility gradients between particles [19,20,21,22]. In highly concentrated systems (>10% *w*/*w*), supersaturation phenomena during storage or processing can lead to recrystallisation, which impairs drug release and uniformity [11,23]. This is particularly relevant for AZA, which lies in a medium solubility range (1–33 mg/mL) where solubility-related instability is more pronounced [7,23,24]. Accordingly, a rational selection of stabilisers is of crucial importance. Recent studies have emphasized the value of a combination of steric (e.g., cellulose ether) and electrostatic (e.g., chitosan) stabilisation mechanisms to prevent particle growth, particularly in the stabilisation of drugs with amphiphilic or surface-active properties [25,26,27,28]. In addition, the manufacturing process itself, e.g., wet milling, can also influence the stability of the nanosuspension by increasing viscosity, causing flocculation or promoting surface destabilisation in poorly optimised systems [29,30,31,32,33].

To address the challenges of formulating nanosuspensions of medium soluble drugs, a preliminary stabiliser screening was performed using wet media milling (WMM) to evaluate a wide range of pharmaceutically acceptable polymers and surfactants. The screening included non-ionic, anionic and cationic stabilisers, with particular attention paid to their wetting ability, steric/electrostatic properties and their ability to prevent aggregation during high-energy milling. Among the tested candidates, hydroxypropyl methylcellulose (HPMC), chitosan and their combination proved to be the most promising in terms of particle size reduction efficiency and colloidal stability (Supplementary Materials, Table S1). While HPMC provided effective steric hindrance, chitosan contributed to positive surface charge and electrostatic repulsion, suggesting that a dual stabilisation approach may provide synergistic benefits for AZA nanosuspensions.

Accordingly, the aim of this study was to investigate the stabilisation mechanisms of highly concentrated AZA nanosuspensions (>10% *w*/*w*) prepared by WMM, focusing on the individual and combined effects of HPMC and chitosan. Emphasis was placed on understanding how these stabilisers affect interfacial properties, surface adsorption and colloidal stability and how they interact with AZA at the solid–liquid interface. For this purpose, a combination of wettability tests (contact angle and immersion tests), atomic force microscopy (AFM) and solid-state characterisation techniques (DSC, XRD, FTIR) were used. By elucidating the role of stabiliser type and interaction mechanism, this study provides a scientific basis for the rational development of nanosuspension formulations for AZA, a moderately soluble API. It also lays the foundation for future optimisation through design of experiments (DoE), which will be the focus of subsequent research.

## 2. Materials and Methods

### 2.1. Materials

**Figure 1:**
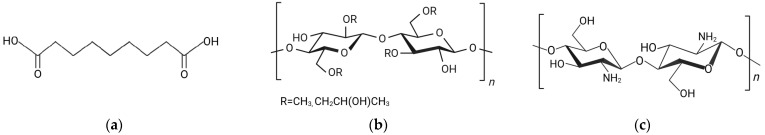
Structural formulas of the main components used in the formulation of AZA nanosuspensions: (**a**) azelaic acid (AZA), (**b**) hydroxypropyl methylcellulose (HPMC) and (**c**) chitosan.

The active pharmaceutical ingredient (API) used in this study was azelaic acid (Dermaz99^®^) from BASF (Ludwigshafen, Germany), with a D90 particle size of 10 µm (Figure 1a). Hydroxypropyl methylcellulose (HPMC) (Methocel^®^ E5 LV) was sourced from DuPont (Wilmington, DE, USA) (Figure 1b), while chitosan (from shrimp shells, low viscosity 20–200 mPas, 1% in acetic acid at 20 °C) was obtained from Sigma Aldrich (Steinheim, Germany) (Figure 1c). Poloxamer 407 (P407) was supplied by Spectrum Chemical (New Brunswick, NJ, USA). Acetic acid and sodium hydroxide (Merck, Darmstadt, Germany) were used for pH adjustment, while purified water served as the dispersion medium. Yttrium-stabilised zirconium oxide beads (Silibeads^®^ type ZY-P Pharma, 0.1–0.2 mm diameter) were procured from Sigmund Linder GmbH (Warmensteinach, Germany) for use in the milling process. All other chemicals and reagents used in this study were of analytical grade.

### 2.2. Preparation of Azelaic Acid Nanosuspensions (AZA-NS)

Azelaic acid (AZA) was dispersed in an aqueous stabiliser solution for 10 min at 600 rpm using a magnetic stirrer (IKA, Staufen, Germany) to form a coarse macrosuspension with an AZA concentration of 5 to 20% (*w*/*w*). The chitosan was pre-dispersed in a 0.1N acetic acid solution before being incorporated into the suspension.

The macrosuspension was then homogenised for 10 min at 3500 rpm using a Silverson LM5 homogeniser (Silverson Machines Inc., East Longmeadow, MA, USA) to break up the agglomerates. The pH of the suspension was adjusted using a 1N sodium hydroxide solution.

Subsequently, 150 g of the macrosuspension was processed with a Dyno-Mill Researchlab agitator bead mill (Willy A. Bachofen AG, Muttenz, Switzerland) at 4000 rpm for 60 min. Yttrium-stabilised zirconium oxide beads (Silibeads^®^ type ZY-P Pharma, Sigmund Linder GmBH, Warmensteinach, Germany) with a size of 0.1–0.2 mm were used for milling. The milling chamber was loaded with 55 mL beads, and the milling was carried out in a circulation process to ensure uniform particle size reduction. As the solubility of AZA is temperature-dependent [34,35], cooling and continuous temperature monitoring were applied throughout the milling process. The composition of the AZA-NS formulations and the milling parameters are summarised in Table 1. All percentages (%) are expressed as *w*/*w*, based on the total weight of the suspension. The relevant formulation parameter is the solid content of AZA (% *w*/*w*), which determines the drug concentration and directly affects the dosing accuracy and colloidal behaviour, which is in line with the principles of carrier-free nano drug systems [36].

### 2.3. Characterisation of AZA-NS

#### 2.3.1. Measurement of Particle Size and Zeta Potential

The particle size distribution and zeta potential of the nanosuspensions were analysed using dynamic light scattering (DLS) with a Zetasizer Nano ZS (Malvern Instruments, Malvern, UK). The particle size distribution results are expressed as intensity-weighted mean particle diameter (Z-average) and polydispersity index (PDI), which reflects the variability of the size distribution.

Prior to measurement, the nanosuspensions were diluted 50-fold (adjusted to the drug content) with a saturated AZA solution, previously filtered through a 0.1 μm membrane filter. All measurements were performed at 25 °C and a backscattering angle of 173°. The mean values were calculated from three independent measurements.

In addition, the nanosuspensions were analysed using an Olympus BX51 optical microscope with 10× to 50× magnification and a QICAM video camera (Olympus, Tokyo, Japan) to assess the presence of microcrystals or agglomerates and to monitor crystal growth during stability testing.

#### 2.3.2. Rheological Characterisation

The rheological characterisation of the nanosuspensions was carried out with a mechanical rheometer (Anton Paar MCR 302, Anton Paar GmbH, Graz, Austria), equipped with a Peltier temperature control unit P-PTD200 and a cone-plate measuring system (angle 1°, diameter 25 mm, CP25, gap 0.049 mm).

Rotational tests were used to determine both the viscosity at rest and the flow behaviour of the system, expressed as viscosity (ƞ) as a function of the shear rate ($\dot{\gamma }$). The viscosity measurements were carried out in a shear rate range from 0.1 s^−1^ to 10,000 s^−1^. To evaluate the viscoelastic properties of AZA-NS under oscillating conditions, an amplitude sweep test was performed in which the shear strain was systematically increased from 0.01% to 100% while maintaining a constant angular frequency of 10 rad/s. Prior to measurement, the sample was allowed to recover its structure for 1 min.

Data analysis, including viscosity (ƞ), shear rate ($\dot{\gamma }$), storage modulus (G′) and loss modulus (G″), was performed using RheoCompass 1.30 software (Anton Paar GmbH, Graz, Austria). All rheological tests were carried out in duplicate at 25 °C.

#### 2.3.3. Short-Term Stability Study

The short-term physical stability of the nanosuspensions was evaluated by analysing the key physical properties after 10 days of storage under accelerated conditions at 40 °C. The parameters evaluated included appearance, Z-average particle size, polydispersity index (PDI), microscopic appearance, zeta potential and redispersibility.

#### 2.3.4. Morphological Characterisation

Atomic force microscopy (AFM) was used to characterise the morphological appearance of the drug nanocrystals and to obtain additional information on the actual crystal size for comparison with the hydrodynamic diameter (Z-average) determined by DLS.

For AFM analysis, the AZA-NS samples were diluted 50-fold with a saturated AZA solution, previously filtered through a 0.1 µm membrane filter, and mounted on mica discs (Highest Grade V1 AFM Mica Discs, Ted Pella Inc., Redding, CA, USA). The samples were then dried under vacuum before imaging.

Morphological analysis was performed using an atomic force microscope NTEGRA Prima (NT-MDT) in intermittent-contact AFM mode. Rectangular cantilevers (NT-MDT NSGO1 silicon, N-type, antimony-doped, with Au reflective coating) with a nominal force constant of 5.1 N/m and a drive frequency of 150 kHz were used. The AFM images were recorded and analysed with the software Gwyddion 2.60 (Free and Open Source Software, Czech Metrology Institute).

Multiple measurements were performed for each sample, covering scan areas of (10 × 10) µm and (5 × 5) µm. Particle profiles were measured to evaluate the morphology and size distribution of the nanosuspension.

### 2.4. Solid-State Characterisation of AZA-NS

#### 2.4.1. Contact Angle and Immersion Time

To measure the contact angle, 150 mg of AZA was pressed into a 13 mm pellet using a hydraulic hand press (Specac, Pleasantville, NY, USA). The Young–Laplace contact angles of the AZA pellets and the aqueous stabiliser solutions were measured with a Krüss drop shape analyser DSA100 (Krüss GmbH, Hamburg, Germany) at first contact and after equilibrium (approximately 30 s after deposition) using the sessile drop technique at room temperature (22 ± 2 °C). Liquid drops of 2 μL were dispensed at a rate of 100 µL/min using a Hamilton syringe onto AZA pellet surface. Each sample was measured six times, and the results are expressed as mean ± SD. Drop shape analysis, including baseline and drop profile detection, and contact angle determination were performed using DSA4 software (Krüss GmbH, Hamburg, Germany).

The immersion time (or sink time test) was performed according to the method described by Binks et al. [37]. In brief, 50 mg of AZA (previously homogenised with a mortar and pestle) was carefully sprinkled from a fixed height onto a liquid surface of stabiliser solution. The time required for the powder to disappear from the liquid surface completely was recorded as the immersion time.

#### 2.4.2. Attenuated Total Reflection Fourier Transform Infrared Spectroscopy (ATR-FTIR)

ATR-FTIR analyses were performed on pure AZA powder, stabilisers (HPMC, chitosan, P407), their physical mixtures (at the same ratio as in the AZA-NS) and AZA-NS samples to evaluate possible drug–stabiliser interactions. FTIR spectra were recorded in the range of 400–4000 cm^−^¹ with a resolution of 4 cm^−1^ using a Tensor II FTIR spectrometer (Bruker, Billerica, MA, USA) equipped with a diamond ATR crystal. Background subtraction was performed, and each spectrum was recorded with 16 scans.

#### 2.4.3. Differential Scanning Calorimetry (DSC)

DSC analyses were performed using a Discovery DSC 2500 Differential Scanning Calorimeter (TA Instruments, New Castle, DE, USA). Precisely weighed 1–2 mg samples were placed in standard aluminium dishes and heated at a rate of 10 °C/min from 25 °C to 200 °C under a nitrogen purge gas flow of 50 mL/min. An empty aluminium dish served as a reference.

The melting temperature depression was calculated as the difference between the onset melting temperatures of pure AZA, physical AZA–stabiliser mixtures and dried AZA-NS samples. The relative degree of crystallinity (RDC) was determined using the following Equation (1):

(1) $$\mathrm{RDC}\left(\%\right)=\frac{{∆H}_{mix}}{{∆H}_{AZA}\times \omega_{AZA}}\times 100$$

where ∆*H_mix_* is the enthalpy of fusion of the sample, ∆*H_AZA_* is the enthalpy of pure AZA and *ω_AZA_* is the actual AZA content in each formulation.

#### 2.4.4. X-Ray Diffraction (XRD)

X-ray diffraction patterns were recorded using an X’Pert Pro diffractometer (PANalytical, Almelo, The Netherlands) equipped with a diffraction beam monochromator that generated CuKα radiation. Scans were performed in the range of 3–40° 2θ with a step size of 0.017° and a scan time of 102 s per step.

#### 2.4.5. Atomic Force Microscopy (AFM)

AFM was used to study the interactions between stabilisers and the AZA surface. The methodology was adopted from Verma et al. [38]. For the analysis, 150 mg of AZA was pressed into 13 mm pellets using a manual hydraulic press (Specac, Orpington, UK) at a pressure of 5 tonnes for 2 min. The production and incubation of the pellets was optimised based on a preliminary screening study in which surface roughness was evaluated under different pressing conditions (mica vs. stainless steel) and incubation temperatures (room temperature vs. refrigeration). The most uniform surface was obtained with pellets pressed by stainless-steel stamps and incubated under chilled conditions to minimise the dissolution of AZA, considering its temperature-dependent solubility.

The prepared pellets were incubated in stabiliser solutions saturated with AZA at 2–8 °C for 10 min. The stabiliser solutions contained 1.25% P407, 0.2% chitosan, 1.5% HPMC and a combination of 0.2% chitosan and 1.5% HPMC. After incubation, the pellets were rinsed with filtered saturated AZA solution and dried under a stream of nitrogen.

For comparison, blank pellets were prepared and treated with filtered saturated AZA solution only. Surface topography and phase images were recorded using an NTEGRA Prima atomic force microscope (NT-MDT) in intermittent contact mode under the same conditions described in Section 2.3.4.

#### 2.4.6. Statistical Analysis

All data are expressed as mean ± standard deviation (SD). Statistical comparisons between two groups were performed using Student’s *t*-test, while comparisons between multiple groups were performed using one-way ANOVA followed by Dunnett’s post-hoc test. A *p*-value of ≤0.05 was considered to indicate statistical significance.

## 3. Results

The particle size of AZA was effectively reduced by WMM, with variations in stabilisers affecting the final particle properties of the AZA-NS samples. Samples with different stabilisers showed different behaviour during milling, ranging from foaming and phase separation at low AZA concentrations (AZA5-C0.1) to problems with processability due to gelation of the suspension (AZA10-H_3.4). Chitosan-based formulations were limited to a maximum AZA concentration of 5%, whereas HPMC allowed drug loading up to 10% AZA at a pH of 3.4, but this led to clogging over time due to the increase in viscosity. Adjusting the pH to 4.5 reduced viscosity. allowing a 20% AZA loading and a milling duration of 1 h. The combination of chitosan and HPMC resulted in an easy-to-process 20% suspension with no signs of clogging, even after extended milling times.

### 3.1. Physicochemical Properties and Short-Term Stability of AZA-NS

The initial physicochemical properties of AZA-NS formulations and their stability after 10 days of storage at 40 °C were evaluated by monitoring Z-average particle size, polydispersity index (PDI), zeta potential, crystal growth on the microscopic image and redispersibility. The results are shown in Table 2.

On day 0, all formulations showed Z-average values below 900 nm, confirming the successful formation of nanosuspensions. The formulations AZA10-H_3.4, AZA20-H and AZA10-HC exhibited uniform particle sizes (~539–541 nm) with low PDI values (≤0.23), indicating narrow size distribution. In contrast, AZA10-H exhibited a larger initial particle size (834.8 nm) and a broader size distribution (PDI = 0.284), suggesting reduced milling efficiency. The formulation AZA20-HC demonstrated the smallest Z-average (326.8 nm) and the lowest PDI (0.208), reflecting a more monodisperse system. The chitosan-only stabilised formulation, AZA5-C0.1, exhibited a slightly larger particle size (554.2 nm) with a wider PDI (0.323).

Zeta potential values on day 0 revealed substantial differences in electrostatic stabilisation among the formulations. HPMC-based formulations (AZA10-H_3.4, AZA10-H, AZA20-H) showed low negative zeta potential values (−2.85 to −4.35 mV), indicating minimal electrostatic repulsion, probably due to the non-ionic nature of HPMC and the limited ionisation of AZA at the pH values tested. Low zeta potentials previously reported for purely sterically stabilised systems do not necessarily reflect a loss of stability and should be carefully evaluated in the context of steric stabilisation mechanisms [39]. The highest zeta potential was observed for AZA5-C0.1 (+39.1 mV), which could be attributed to the protonated amine groups of chitosan leading to a strong positive surface charge. In contrast, dual-stabiliser formulations containing both HPMC and chitosan (AZA10-HC, AZA20-HC) showed moderately positive zeta potential values (from +12.0 to +18.0 mV), which may have resulted from partial charge neutralisation by HPMC and enhanced steric stabilisation [40,41].

Following 10 days of storage at 40 °C, notable differences in physical stability of AZA-NS formulations were observed. Formulations AZA10-H and AZA5-C0.1 exhibited pronounced particle growth, with Z-average values increasing to 1205 nm and 803.9 nm, respectively, accompanied by a marked rise in PDI. These formulations also showed significant shifts in zeta potential, reaching +7.81 mV for AZA10-H and +30.5 mV for AZA5-C0.1, indicating possible aggregation. In contrast, HPMC-stabilised formulations (AZA10-H_3.4, AZA20-H) maintained relatively stable particle sizes (~555–613 nm) and PDI (<0.30), confirming their stability over time. The dual-stabilised formulations AZA10-HC and AZA20-HC showed a moderate increase in particle sizes, however the final values (414.9–613.9 nm) remained well within the nanometre range, confirming effective stabilisation. All AZA-NS samples retained good redispersibility, appearing homogeneous after vigorous shaking for 10 s, with no visible signs of caking or agglomeration.

Statistical analysis was performed to assess the significance of changes between day 0 and day 10 for each stability-indicating parameter. Paired *t*-tests revealed significant differences (*p* < 0.05) in the Z-average and PDI values, particularly for AZA10-H and AZA5-C0.1, which showed the greatest increase in particle size. The changes in zeta potential were also significant for AZA10-H (*p* < 0.05) and AZA5-C0.1 (*p* < 0.01), which correlates with the observed aggregation. A one-way ANOVA performed on day 10 data confirmed statistically significant differences between formulations (*p* < 0.01), suggesting that stability is formulation dependent. Post-hoc comparisons showed that AZA10-HC and AZA20-HC had a significantly lower increase in particle size compared to AZA5-C0.1 and AZA10-H (*p* < 0.01), supporting the stabilising effect of HPMC–chitosan combinations.

### 3.2. Rheological Characterisation

**Figure 2:**
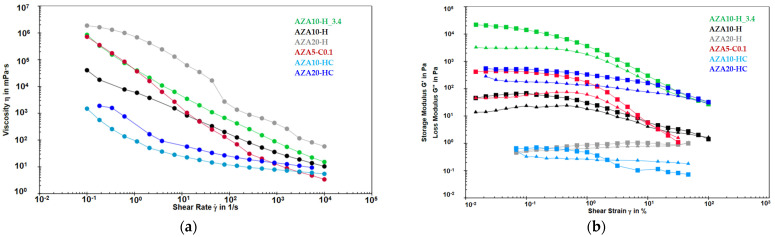
(**a**) Flow curves of AZA-NS formulations showing viscosity (η) as a function of shear rate (γ). All formulations show shear thinning behaviour, with viscosity varying depending on the type and concentration of stabiliser. (**b**) Amplitude sweep test results showing the storage modulus (G′), represented by squares, and the loss modulus (G″), represented by triangles, as a function of shear strain.

The rheological properties of the AZA-NS formulations were evaluated to assess their viscosity profiles, flow behaviour and viscoelastic properties. Flow curves (viscosity versus shear rate) and amplitude sweep results (storage and loss modulus versus shear strain) are presented in Figure 2a and Figure 2b, respectively.

All formulations exhibited shear-thinning (pseudoplastic) behaviour, characterised by a decrease in viscosity with increasing shear stress. This indicates the presence of structured dispersions that undergo progressive structural degradation under the applied shear stress. Such rheological behaviour is desirable for topical nanosuspensions, as it facilitates application while maintaining structural stability at rest. Among the tested formulations, AZA20-H (grey) and AZA10-H_3.4 (green) displayed the highest viscosities across the entire range of shear rates, particularly at low shear rates, suggesting a stronger internal network compared to the other formulations. Sample AZA10-H (black) exhibited an intermediate viscosity, with a substantially lower viscosity at low shear rates but less pronounced differences in viscosity at high shear rates compared to AZA10-H_3.4 (green). The samples differed only in pH.

Formulations containing both HPMC and chitosan (HC variants, blue and light blue) demonstrated significantly lower viscosities than their HPMC-only counterparts, confirming that the combined stabilisation mechanism resulted in a weaker intermolecular structure. Among these, AZA10-HC (light blue) consistently exhibited the lowest viscosity, indicating higher flowability and lower shear deformation resistance.

AZA5-C0.1 (red) initially exhibited a viscosity comparable to AZA10-H_3.4 (green) at low shear rates. However, it showed the most pronounced shear thinning effect with the fastest viscosity decrease of all formulations tested and finally reached the lowest viscosity at high shear rates. This indicates that the stabilisation by chitosan in this formulation formed a weak structure that was very susceptible to degradation with increasing shear stress.

The storage modulus (G′) and loss modulus (G″) of AZA-NS stabilised with HPMC, chitosan or their combination were evaluated as a function of shear strain (γ) (Figure 2b). The amplitude sweep test provides information on the formulation’s viscoelastic properties, flow behaviour and network stability.

At low shear strain (γ < 1%), all formulations exhibited a linear viscoelastic region (LVR) in which the applied stress had little effect on the internal structure of the nanosuspensions. The storage modulus (G′) reflected the elastic component of the viscoelastic behaviour and indicated the solid-state properties of the sample. In contrast, the loss modulus (G″) reflected the viscous component of the viscoelastic behaviour. For all samples, G′ was higher than G″ at low strains, confirming that the formulations predominantly behaved as weak gels or viscoelastic solids. AZA10-H_3.4 (green) had the highest G′ value, confirming the strongest internal structure, probably due to the lower pH (3.4), which improved particle interactions and network density. AZA20-HC (dark blue) and AZA5-C0.1 (red) followed closely behind, indicating comparable viscoelasticity. AZA10-H (black) showed a lower but still considerable G′, while AZA20-H (grey) and AZA10-HC (light blue) showed the weakest elastic networks, suggesting lower structural integrity. Notably, AZA5-C0.1 (red) had the largest initial difference between G′ and G″, indicating that while the elastic component was high, the viscous contribution was also substantial. This indicates a more pronounced viscoelastic behaviour, possibly due to weaker structuring or greater molecular mobility in the system.

With increasing shear strain, all reached reach a critical point at which G′ began to decrease, marking the beginning of the structural collapse. The point at which G′ and G″ intersected (gel-to-sol transition point) represents the yield point at which the material transitioned from a predominantly elastic to a more viscous state. AZA10-H_3.4 (green) had the highest yield point, confirming its superior resistance to deformation. AZA20-HC (dark blue) and AZA10-H (black) gad similar yield points, which were slightly lower than those of AZA10-H_3.4, indicating moderate structural strength. AZA5-C0.1 (red) reached its yield point earlier, indicating a weaker network compared to the higher-rated formulations. AZA20-H (grey) and AZA10-HC (light blue) had the lowest yield points, confirming that these formulations lose their elastic properties at lower strains. AZA20-H (grey) showed irregular fluctuations of G′, indicating microstructural restructuring under shear stress.

### 3.3. Morphological Characterisation

**Figure 3:**
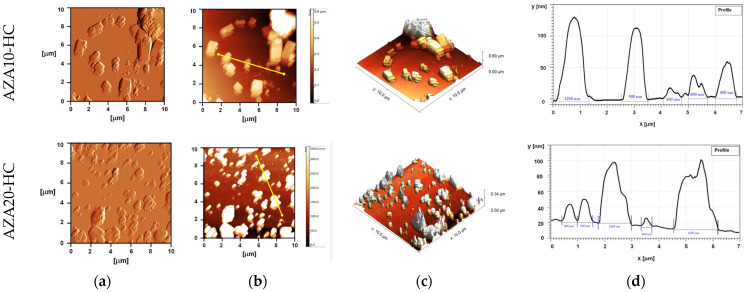
Atomic force microscopy (AFM) analysis of AZA-NS formulations. (**a**) Error signal showing the surface morphology and particle size distribution of AZA10-HC (top) and AZA20-HC (bottom). (**b**) 2D topography showing differences in particle size and aggregation. (**c**) 3D topography illustrating variations in roughness. (**d**) Height profiles illustrate differences in particle size.

AFM analysis of AZA-NS stabilised with a dual stabiliser system provided detailed insights into morphology of the AZA nanocrystals, particle size and aggregation behaviour (Figure 3). AZA10-HC exhibited larger, well-defined crystalline structures, while AZA20-HC showed a more homogeneous distribution of smaller nanocrystals accompanied by irregular aggregates. Both formulations exhibited a platelet-like morphology, with AZA20-HC containing a higher density of crystals per unit area, consistent with its higher AZA concentration.

The height and diameter profiles from AFM scans supported these results, showing particle heights of approximately 100 nm with diameters ranging from 650 to 1250 nm in AZA10-HC, while AZA20-HC exhibited a lower surface roughness with particle diameters predominantly between 400 and 1600 nm.

However, the particle size values obtained from the AFM height profiles (Figure 3d) were significantly larger than the hydrodynamic values (Z-average from DLS, Table 2). These differences are likely due to the aggregation of particles during the drying process required for AFM sample preparation, resulting in the stacking structures visible in the 3D topography images (Figure 3c). In addition, the DLS assumes a spherical geometry, which may underestimate the dimensions of the platelet-shaped AZA nanocrystals. Previous studies [42] have confirmed this and have shown that the Z-average values correlate with the particle width, while the actual particle lengths measured with AFM can be considerably larger. Therefore, diagonal measurements captured by AFM height profiles may further contribute to the apparent increase in particle size.

### 3.4. Investigation of the Formability and Stabilisation Mechanisms of AZA-NS

Representative formulations containing HPMC, chitosan, their combination and Poloxamer^®^ 407 (P407) were systematically analysed to investigate the interactions responsible for the formation and stability of the AZA nanosuspensions. To gain deeper insights into the underlying mechanisms, the study also included the P407-based formulation (AZA5-P), which failed to form a nanosuspension (Z-average: 1100 nm, Supplementary Information, Table S1), so that a comparative evaluation of the effectiveness of the stabilisers was possible.

#### 3.4.1. Wettability

The wettability of AZA in aqueous stabiliser solutions was evaluated using contact angle measurements and immersion time tests as shown in Table 3. These tests provide insight into the ability of different stabilisers to facilitate the wetting and dispersion of AZA particles, which is key for the successful formulation of nanosuspensions (AZA-NS). According to the established wetting principles, equilibrium contact angles (ECA) below 90° facilitate spontaneous immersion, while those above 90° require additional work for complete wetting [23,37,43,44]. However, more recent studies have suggested that the threshold value for the definition of hydrophilicity should be corrected to 43°, as surfaces with an ECA value above this value may still exhibit partial non-wetting behaviour [45].

The tests were carried out for different types of stabilisers at the highest concentrations used in the preliminary milling trials. The results for all stabilisers tested are shown in Table S1 (Supplementary Material), while Table 3 shows representative results for HPMC, chitosan and their combination. For comparison, the wettability of AZA in purified water and P407 solution, which did not enable nanosuspension formation, is also shown.

All stabiliser solutions tested exhibited equilibrium contact angles (ECA) above 43°, indicating that AZA has a partially hydrophilic nature with moderate wettability. However, significant differences were found between the stabilisers.

The combination of 1.5% HPMC and 0.2% chitosan resulted in the highest initial contact angle (77.8 ± 4.9°) but also the strongest decrease over time, reaching the lowest equilibrium contact angle (45.8 ± 3.1°). This indicates that the synergistic effect of HPMC and chitosan significantly improves the wettability of AZA, probably due to enhanced surface interactions. In contrast, 0.2% chitosan alone resulted in a significantly higher ECA value (56.4 ± 3.7°) and poor immersion behaviour as the AZA powder remained suspended for more than one hour, confirming its limited ability to improve wettability. The 1.5% HPMC solution had a lower ECA value (51.7 ± 4.0°) compared to chitosan alone and allowed for immediate immersion, suggesting that HPMC facilitates rapid initial wetting despite an ECA value above 43°.

A 1.25% P407 solution had an ECA value of 48.2 ± 2.1°, comparable to the HPMC–chitosan combination. However, despite its promising wetting properties, P407 could not help to reduce the particle size of AZA to nanodimensions using WMM.

For comparison, AZA in purified water had an ECA of 51.0 ± 2.1° and was not completely wetted within one hour, confirming that water alone does not provide sufficient wetting for AZA particles.

The immersion time test further confirmed these trends. AZA powder in HPMC, HPMC–chitosan and P407 solutions immersed immediately. In contrast, AZA in water and chitosan solutions remained floating for extended period, confirming the poor wettability of chitosan when used alone. These results suggest that HPMC improves the wettability of AZA compared to water and chitosan, while the HPMC–chitosan combination provides the most effective surface wetting, probably due to complementary steric and electrostatic interactions.

#### 3.4.2. Intramolecular Interactions, Thermal Properties and Crystallinity

**Figure 4:**
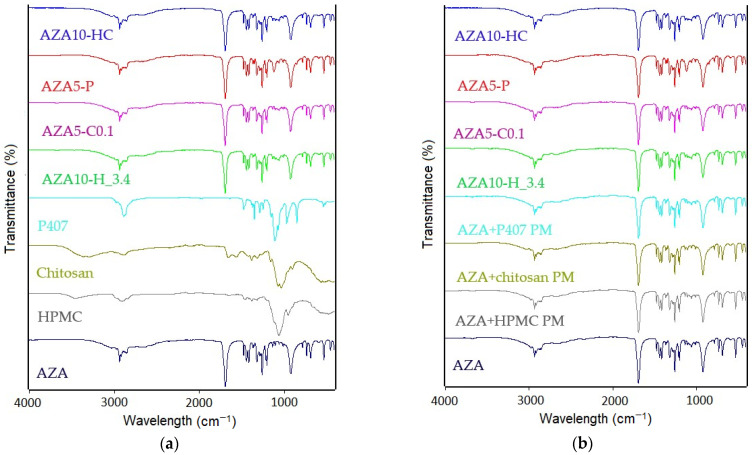
Fourier transform infrared spectroscopy (FTIR) analysis of azelaic acid (AZA) nanosuspensions and their constituents. (**a**) FTIR spectra of pure AZA, individual stabilisers (HPMC, chitosan, P407), AZA-NS formulations (AZA10-H_3.4, AZA5-C0.1 and AZA10-HC) and the suspension formulation with P407 (AZA5-P), showing the retention of the structural integrity of AZA. (**b**) Comparison of AZA-NS and AZA5-P with the corresponding physical mixtures (PM) showed no major spectral shifts, confirming that the stabilisation of the nanosuspension was primarily due to physical adsorption rather than chemical interactions.

FTIR analysis was performed to investigate possible molecular interactions between AZA and the representative stabilisers in nanosuspension formulations. Figure 4a shows FTIR spectra of pure AZA, individual stabilisers (HPMC, chitosan, P407), AZA-NS formulations (AZA10-H_3.4, AZA5-C0.1 and AZA10-HC) and a sample containing P407, designated AZA5-P. In Figure 3b, the spectra of AZA-NS and AZA5-P are compared with the corresponding physical mixtures (PM) prepared in formulation-equivalent ratio, which provides information about possible interactions during the milling process and AZA-NS formation. FTIR spectra of physical mixtures in 1:1 ratio are included in the Supplementary Materials (Figure S1).

The characteristic peaks of AZA were observed at 1684 cm^−1^, corresponding to the C=O stretching vibration of the carboxyl groups, and 1251 cm^−1^, related to the C–O stretching vibration, in agreement with previous reports [17,46,47]. These peaks were present in all AZA-NS formulations, indicating that AZA retained its structural integrity.

The spectra of all AZA-NS samples and AZA5-P showed no significant shifts in the characteristic AZA peaks, indicating that no strong chemical interactions between AZA and these stabilisers occurred.

A DSC analysis was carried out to evaluate the thermal behaviour of pure ingredients, AZA-NS and the corresponding physical mixtures. Table 4 shows the main thermal parameters, including the melting point (MP), onset temperature, enthalpy (J/g), relative degree of crystallinity (RDC) and melting point depression (MP depression) for each formulation. DSC thermograms are included in the Supplementary Materials (Figures S3 and S4). The DSC analyses confirmed the amorphous nature of chitosan and HPMC, as shown by the absence of distinct melting peaks in the thermograms. P407 showed a clear endothermic melting peak at 56.02 °C, indicating its crystalline structure.

The DSC thermogram of pure AZA showed a characteristic endothermic peak with an onset temperature of 107.01 °C and an enthalpy of fusion of 204.09 J/g, which is consistent with previously reported values [17,34,48]. In comparison, all physical mixtures showed a slight decrease in melting point with minimal effects on crystallinity, with the exception of the AZA P-407 PM, which showed a significant decrease in crystallinity.

Among the AZA-NS formulations, AZA10-H_3.4 showed a moderate melting point depression (−3.05 °C) with a largely preserved crystalline structure, while AZA5-C0.1 exhibited the most pronounced melting point depression (−8.85 °C), indicating a strong interaction between chitosan and AZA. The combination of HPMC and chitosan in AZA10-HC resulted in an intermediate melting point decrease (−6.78 °C with a slight decrease in crystallinity, confirming a synergistic stabilisation effect). The RDC values followed a similar trend, with AZA-HPMC formulations exhibiting a higher degree of crystallinity than chitosan-containing formulations. In particular, the AZA-P407 formulation (AZA5-P) showed a significant loss of crystallinity with a reduction in enthalpy to 139.99 J/g and an RDC of 85.74%, suggesting increased AZA amorphisation or miscibility with the melted polymer. Moreover, DSC thermograms revealed the formation of a eutectic mixture between AZA and P407 at a 1:1 weight ratio (Supplementary Materials, Figure S3).

**Figure 5:**
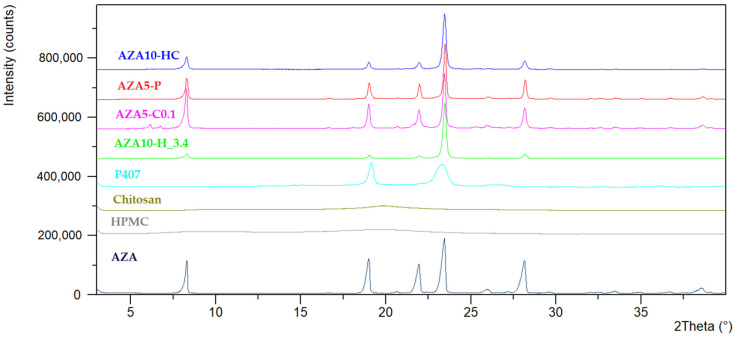
X-ray diffraction (XRD) patterns of pure AZA, HPMC, chitosan, P407, AZA-NS formulations (AZA10-H_3.4, AZA5-C0.1 and AZA10-HC) and AZA P407 suspension (AZA5-P).

The X-ray diffraction (XRD) patterns of AZA, pure excipients and AZA-NS stabilised with different excipients are shown in Figure 5. XRD diffractograms of corresponding physical mixtures are provided in the Supplementary Materials (Figure S2).

The diffraction patterns show that all AZA-NS samples exhibited the characteristic diffraction peaks of crystalline AZA, with the most intense peak observed at ~24° 2θ, which remained unchanged in position and intensity in all formulations. This indicates that the primary crystalline structure of AZA was preserved in all nanosuspensions, and there was no evidence of polymorphic transformation based on the observed XRD pattern. XRD diffractograms of chitosan, HPMC and P407 are in line with the DSC thermograms, confirming crystallinity only for P407.

The order of reduction of the intensity of the secondary peaks was as follows: AZA5-C0.1 > AZA5-P > AZA10-HC > AZA10-H_3.4. The lack of peak broadening in all samples, despite a known reduction in particle size, suggests that the nanocrystals fell within a size range where size-induced peak broadening was minimal. Therefore, the observed decrease in peak intensity is more likely attributable to a partial loss of crystallinity, rather than to changes in crystal structure or particle size effects [49]. These findings indicate that the extent of the reduction in peak intensity depends on the stabiliser used, with HPMC and low pH (AZA10-H_3.4) leading to the greatest reduction. At the same time, chitosan alone (AZA5-C0.1) had the least effect on the crystalline order of AZA and even showed an increase in intensity for some peaks.

#### 3.4.3. Surface Interactions Between AZA and Stabilisers

**Figure 6:**
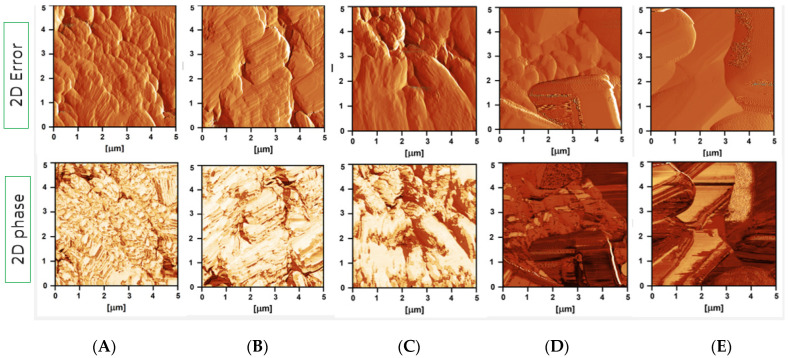
Atomic force microscopy (AFM) images of AZA pellets incubated in stabiliser solutions. 2D error signal images (top row) and 2D phase images (bottom row) of azelaic acid (AZA) pellets incubated in stabiliser solutions: (**A**) 1.5% HPMC, (**B**) 0.2% chitosan, (**C**) 1.5% HPMC + 0.2% chitosan, (**D**) 1.25% P407 and (**E**) saturated AZA solution (control).

Atomic force microscopy (AFM) was used to investigate the surface coverage of AZA pellets incubated in different stabiliser solutions. The 2D error images provide insight into the topographical features of the AZA surface, while the 2D phase images provide an additional visualisation of stabiliser adsorption (Figure 6).

The phase images in Figure 6 show clear differences in stabiliser adsorption between the formulations tested. Pellets incubated in 1.5% HPMC (A), 0.2% chitosan (B) and a combination of 1.5% HPMC and 0.2% chitosan (C) displayed pronounced surface coverage, indicated by the extensive presence of light-coloured areas. Sample (C), treated with a mixture of HPMC and chitosan, displayed a combination of localized bright and less intense regions, suggesting the adsorption of stabilizers to a greater extent on exposed crystal surfaces while being less prominent in the crevices. The absence of clear distinction between the two polymers in the phase images likely resulted from their high miscibility or similarities in viscoelastic and adhesive properties, which may have resulted in a uniform phase response.

In contrast, the pellets treated with 1.25% P407 (D) showed only scattered bright areas, indicating poor adsorption of the stabiliser and an uneven surface coating. The control sample (E), which was incubated in a saturated AZA solution, showed no significant bright areas, confirming the lack of adsorption in the absence of polymeric excipients.

## 4. Discussion

The successful stabilisation of AZA-NS requires a comprehensive understanding of the interactions between the active pharmaceutical ingredient and the selected stabilisers, as well as their influence on crystallinity, rheological behaviour and morphological characteristics. The findings of this study demonstrate that the combination of HPMC and chitosan provides a dual stabilisation mechanism that improves the physical stability, processability and wettability of AZA-NS. When used individually, both stabilisers presented certain limitations. Chitosan provided electrostatic stabilisation and was very effective in reducing AZA particle size. However, during wet media milling, it promoted foam formation, leading to processing challenges and restricting its concentration to 0.1%, which, in turn, limited the achievable drug loading to 5% AZA. On the other hand, HPMC significantly increased viscosity of the dispersion, especially at acidic conditions (pH 3.4), resulting in gel-like systems that impaired milling efficiency and attainment of particle sizes optimal for targeting the follicular route in dermal drug delivery.

The increase in viscosity in WMM is a known challenge in the development of nanosuspensions [33,50]. As the particle size decreases, the number of particles per unit volume rises, which increases the interactions between the particles—especially in the submicron range. Polysaccharide stabilisers such as HPMC and chitosan further contribute to viscosity by forming a hydration layer around the nanoparticles, which can impede fluid flow and promote gelation [51].

Although both polymers are widely used in pharmaceutical research and formulation development, their combined application in nanosuspensions, especially for medium soluble drugs, is still poorly understood. HPMC, a steric stabiliser, forms a protective layer around the drug particles that prevents aggregation and sedimentation [43,52]. Chitosan, a cationic polysaccharide, provides both steric and electrostatic stabilisation through interactions with negatively charged drug surfaces, thus enhancing colloidal stability [53].

The results of this study confirm that the stabilisation of AZA, a medium soluble drug, requires a synergistic effect of steric stabilisation by HPMC and electrostatic stabilisation by chitosan. This dual stabilisation strategy effectively modulated drug–stabiliser interactions to maintain colloidal stability without excessive increase in solubility, thus preventing unintended recrystallisation and phase separation that could lead to instability [24,54].

### 4.1. Physico-Chemical Characterisation and Stability of AZA-NS

WMM enabled the preparation of AZA-NS with particle sizes between 400 and 700 nm—an optimal range for enhanced dermal penetration and prolonged skin retention [4]. Several formulation parameters, including pH, viscosity, stabiliser type and concentration and active ingredient loading, significantly influenced the final particle size and distribution (Table 2).

Maintaining a pH of 4.5 was found to optimise drug solubility and ionisation, enhance stabiliser interactions and minimise excessive dissolution, while simultaneously enabling electrostatic stabilisation by chitosan [40]. This pH value also supports the homeostasis of the skin and inhibits the growth of *Cutibacterium acnes* [12,55].

Chitosan-stabilised nanosuspensions (~554 nm) exhibited high positive zeta potential (+39.1 mV), suggesting effective electrostatic stabilisation. However, the appearance of needle-like crystals (~10 µm) indicated early destabilisation and potential recrystallisation [6,52]. HPMC-based nanosuspensions yielded particles between 541 and 834 nm with low PDI (0.22–0.28), while the addition of chitosan further reduced the particle size (326–541 nm) and increased the zeta potential (12–18 mV), indicating synergistic steric-electrostatic stabilisation [56]. Ionic surfactants stabilise nanosuspensions primarily by electrostatic repulsion, with zeta potential values around ±30 mV generally associated with stable systems. However, in systems containing steric stabilisers such as HPMC, adsorption of the polymer leads to a shift in the shear plane and a reduction in the measured zeta potential, which should not be misinterpreted as reduced stability [11,39].

Higher drug loading was also found to enhance particle size reduction efficiency. The AZA20-HC achieved significantly smaller Z-average (326.8 ± 2.60 nm, *p* < 0.05), likely due to increased particle collisions during WWM at higher solid content [40,57]. Similar trends have been observed in other studies on nanosuspensions, where drug concentration has played a key role in optimising particle size and has influenced dissolution and pharmacokinetic performance [58,59].

The short-term stability study demonstrated that nanosuspensions stabilised with HPMC alone (AZA10-H, AZA20-H) or in combination with chitosan up to a 10% drug loading (AZA10-HC) maintained their nanoscale particle size with minimal growth after 10 days of storage at 40 °C (Table 2). In contrast, the formulation with only chitosan (AZA5-C0.1) showed significant particle growth, likely due to insufficient steric stabilisation at the low chitosan concentration (0.1%). These results suggest that HPMC plays a critical role in steric stabilisation, whereas chitosan alone is less effective in preventing particle aggregation Although the AZA20-HC formulation initially showed the most efficient particle size reduction, it exhibited marked crystal growth after 10 days of storage at 40 °C (Table 2). This instability may have resulted from the supersaturation effect and increased dissolution rate of smaller particles at high drug concentrations, which promote recrystallisation and crystal growth [60,61]. In the case of AZA, these phenomena were further exacerbated by its temperature-dependent solubility. Therefore, optimisation of the HPMC-to-chitosan ratio and overall polymer concentration is essential to improve the long-term stability of 20% AZA nanosuspensions.

The results presented show that the optimisation of the stabiliser system, particle size and drug loading is crucial for balancing immediate processability and long-term stability of nanosuspensions of medium soluble drugs.

### 4.2. Rheological Properties and Processability

The viscosity profiles (Figure 2a) revealed that all AZA nanosuspensions exhibited shear-thinning (pseudoplastic) behaviour, a desirable property for dermal formulations as it allows for ease of application and improved spreadability [3]. This behaviour can be attributed to the progressive disruption of the interactions between the particles and the polymer networks under increasing shear stress. However, significant differences in viscosity trends were observed among the formulations, highlighting the influence of AZA concentration, pH and stabiliser type.

A higher AZA concentration (10% vs. 20%) resulted in higher viscosity at low shear rates, indicating greater structural integrity due to intensified particle–particle interactions. However, this effect was less pronounced at high shear rates, likely due to structural rearrangements and possible agglomeration effects. These findings are consistent with those reported by Pinar et al. [54], who demonstrated that nanosuspension viscosity was strongly dependent on the polymer content and drug–polymer interactions. As particle size decreases, surface-related interactions—such as electrostatic forces, hydration layer formation, and potential aggregation—become more prominent, leading to increased viscosity. Additionally, higher solid content further contributes to structure formation and viscosity enhancement. Polymeric stabilisers with a molecular weight of more than 1000 kDa increase viscosity due to hydrogen bonding with the particles and polymeric entanglements [31].

A lower pH (3.4) further increased viscosity, likely due to altered electrostatic interactions that may affect particle stabilisation and polymer conformation. This was most evident in the formulation AZA10-H_3.4, which displayed highest viscosity among all samples. Adjusting the pH to 4.5 reduced the extent of hydrogen bonding between partially ionised AZA (-COO-) and HPMC, which reduced the viscosity and enabled successful milling of HPMC-based AZA nanosuspensions with up to 20% drug loading [51].

Similar findings were reported by Huang et al. [62], who demonstrated that suspension gelation driven by hydrogen bonding was strongly dependent on pH, shear rate and polymer concentration.

The chitosan-stabilised nanosuspension (AZA5-C0.1) exhibited the most pronounced shear-thinning behaviour, indicating weaker structural integrity compared to HPMC-stabilised systems. The combination of HPMC and chitosan (AZA-HC formulations) resulted in a lower viscosity than HPMC alone, suggesting that steric and electrostatic stabilisation mechanisms work together to create a less cohesive system with weaker intermolecular interactions, making the formulation more flowable and easier to process.

The amplitude sweep test (Figure 2b) provided additional information on the viscoelastic properties of AZA-NS. AZA10-H_3.4 exhibited the highest storage modulus (G′), indicating the strongest internal structuring. In contrast, AZA5-C0.1 initially exhibited a relatively high G′, but the rapid decrease under strain indicated a weak network prone to collapse. AZA20-H and AZA10-HC exhibited the lowest G′ values, indicating lower elastic strength and greater susceptibility to deformation under shear stress.

Although smaller particle sizes typically result in higher viscosity due to stronger interparticle interactions, the rheological behaviour in the present study was primarily governed by the properties of the polymeric stabilisers. [44]. This effect was most pronounced in the formulation AZA20-HC, which had the smallest particle size distribution (Z-average: 326.8 ± 2.60) and the highest polymer and active ingredient concentration but showed only a moderate increase in viscosity.

The presented results provide valuable insights for formulation design, especially for optimising the stability, spreadability and application behaviour of nanosuspensions intended for topical and dermatological use [6,63]. In addition, rheological characterisation of highly drug-loaded nanosuspensions such as AZA-NS gives more profound insights into the interactions between particles and polymers, including hydrogen bonding and electrostatic forces, which affect the viscosity, processability and scalability of such systems [44,60].

### 4.3. Investigation of Stabiliser–Drug Interactions

Understanding the interactions between AZA and stabilisers is crucial for developing stable nanosuspensions with optimal properties for dermal delivery. Two key factors influencing these interactions are the wettability of AZA in stabiliser solutions and the physico-chemical changes induced by stabilisers at the molecular level. To investigate these aspects, we used contact angle measurements, immersion time analysis and solid-state characterisation techniques such as ATR-FTIR, DSC, XRD and AFM.

#### 4.3.1. Surface Interactions and Wettability

Contact angle and immersion time measurements provide complementary insights into the wetting behaviour of stabilisers and their ability to coat and disperse AZA nanocrystals. While contact angle measurements reflect the equilibrium properties at the solid-liquid interface, immersion tests evaluate the dynamic wetting behaviour and indicate how efficiently the stabiliser displaces the air and facilitates the penetration of the liquid into the powder bed [37].

The wettability studies showed that the HPMC–chitosan combination provided the most effective surface coverage, as evidenced by the lowest equilibrium contact angle (45.8 ± 3.1°), highest contact angle reduction (32.0 ± 2.7°) and the immediate immersion of the AZA powder. These results suggest a synergistic effect between HPMC and chitosan, probably due to complementary steric and electrostatic interactions that improve surface coverage. In contrast, chitosan alone exhibited poor wettability, as reflected by a higher ECA value (56.4 ± 3.7°) and a longer immersion time, suggesting that chitosan alone is not sufficient to promote efficient dispersion of AZA particles. Similarly, P407, while exhibiting moderate ECA (48.2 ± 2.1°) and immediate immersion, did not support nanosuspension formation, suggesting that wetting alone is not a sufficient predictor of nanosuspension success, and additional stabilisation mechanisms are required. These results are consistent with previous studies showing that stabilisers with high wettability increased the stability of nanosuspensions by promoting stronger drug–stabiliser interactions [51]. Although the differences in contact angle values may appear small, they were statistically significant (*p* < 0.05), and their functional significance was confirmed by the clear differences in immersion time and formulation results. These results underline the importance of interpreting the contact angle measurements in conjunction with the immersion behaviour and performance of the nanosuspension, rather than considering them in isolation.

Phase imaging, an AFM technique, detects changes in surface properties such as adhesion, hardness, viscoelasticity and friction by measuring phase shifts in cantilever vibration [64,65]. It is particularly useful for imaging polymer material surfaces and is therefore ideal for studying the adsorption of polymer stabilisers on drug surfaces, where differences in composition appear as light and dark areas in phase images [66,67].

AFM phase imaging confirmed that HPMC, chitosan and their combination adsorbed effectively on the AZA surface, while P407 showed poor adsorption, with minimal surface interaction, suggesting weak stab. The results obtained were consistent with the milling experiments, where both HPMC and chitosan successfully formed nanosuspensions, while Poloxamer was unsuccessful due to poor interaction and adsorption on the AZA surface. Similar results were obtained by Verma et al. [38], where HPMC adsorbed on the ibuprofen surface in an extended open-chain conformation, promoting better stabilisation, while PVP and Poloxamer showed insufficient adsorption.

#### 4.3.2. Intramolecular Interactions, Thermal Properties and Crystallinity

ATR-FTIR analysis revealed no significant chemical interactions between AZA and the stabilisers, suggesting that stabilisation primarily occurs through physical adsorption rather than covalent binding. This observation is consistent with previous studies showing that polymeric stabilisers such as HPMC and chitosan stabilise nanosuspensions by adsorbing onto the drug surface without altering its chemical structure [68,69]. In particular, HPMC has been shown to stabilise particles via steric hindrance, preventing aggregation and maintaining colloidal stability [70].

DSC analysis revealed a melting point decrease across all AZA-NS formulations, with the most substantial depression observed in chitosan-containing nanosuspensions, indicating increased amorphisation and crystal lattice disorder. A similar effect has been described in the literature, where chitosan was shown to induce structural disorder and reduce crystallinity in nanosuspensions [41]. The combination of HPMC and chitosan further reduced crystallinity, while also contributing to physical and thermal stability. The relative degree of crystallinity (RDC) results followed a similar trend as melting point depression. According to the Gibbs–Thomson equation, nanocrystals have lower melting points due to their small size and larger surface area, an effect further influenced by high-energy processes such as wet milling [22,71]. Patel et al. [72] reported a correlation between melting point reduction, stronger drug–stabiliser interactions and improved colloidal stability, as supported by the Flory–Huggins model.

X-ray diffraction (XRD) analysis confirmed that all nanosuspensions retained the crystalline structure of AZA, as evidenced by the preservation of the dominant diffraction peak at ∼24° 2θ (Figure 5). These results are consistent with previous reports on wet-milling processes, where water was shown to act as a plasticiser and prevent excessive amorphisation due to a lowering of glass transition temperature [73]. However, differences in the intensity of the secondary diffraction peaks were observed, with AZA10-H_3.4 showing the most significant decrease in peak intensity, followed by dual-stabilised formulation AZA10-HC, whereas AZA5-C0.1 showed an increase in peak intensity. The apparent discrepancy between XRPD and DSC results can be explained by the different detection mechanisms. While XRPD detects long-range crystallinity, DSC is more sensitive to possible localised amorphous regions on the crystal surface, changes in molecular mobility and reduced particle size. The apparent increased crystallinity of the chitosan-based sample (increased intensity of the peak at ~8° 2θ) could have been due to crystal morphological changes caused by the milling process leading to overexpression of some crystal planes, as previously reported for nanosuspensions stabilised by polymers [30,49,73]. In addition, the chitosan sample showed signs of early destabilisation due to the formation of needle-shaped crystals, which could have also affected the XRD peak intensities and the overall pattern. In contrast, the reduced peak intensities observed in HPMC-containing formulations did not necessarily indicate amorphisation. Instead, they may have reflected more efficient particle dispersion in the polymer matrix, resulting in a decreased number of coherent diffraction domains. The lack of peak broadening in the XRPD suggests that the crystallite size was above the threshold of 100 nm, at which Scherrer broadening becomes significant [74]. Together, these results emphasise the need to use several complementary analytical techniques to distinguish between true crystallinity loss and polymer-induced stabilisation effects.

#### 4.3.3. Proposed Mechanism of AZA-NS Formation and Stabilization

Successful formation and stabilisation of AZA-NS by HPMC and chitosan can be attributed to a combination of physical phenomena, including the Rehbinder effect, steric and electrostatic stabilisation and hydrogen bonding. The Rehbinder effect, which facilitates the formation of nanoparticles during milling, occurs when surfactants adsorb on crystal defects, reducing the surface energy and weakening the crystal lattice, which promotes fragmentation [75]. The polysaccharide chitosan, positively charged at a pH of 4.5, interacts electrostatically with the partially ionised AZA, resulting in effective crystal disruption and amorphisation [43]. Evidence for the observed Rehbinder effect in chitosan was provided by DSC data, which showed the strongest melting point depression in a chitosan-based formulation, indicating crystal lattice disruption and a surface amorphisation. In addition, the increase in the intensity of the certain XRD peaks in the chitosan-based sample indicated a possible overexpression of the crystal planes exposed during milling due to the breakage of the crystals along the facet with the lowest binding energy. Due to its strong affinity for AZA surfaces, chitosan offers high milling efficiency at low concentrations. However, it does not provide sufficient steric stabilisation to ensure the required level of wetting that prevents phase separation during milling and early destabilisation due to Ostwald ripening.

In contrast, HPMC increases the stability of the nanosuspension by forming a hydrated polymeric layer around the AZA nanoparticles, which effectively reduces Ostwald ripening by restricting molecular diffusion and crystal growth [76,77]. The primary interaction between AZA and HPMC occurs through hydrogen bonding between the methoxy and hydroxypropoxy groups of HPMC and the carboxyl group of AZA, as supported by the rheological characterisation of AZA-NS with HPMC.

Meanwhile, P407, although widely used for stabilising hydrophobic drugs suspensions, has been shown to be less efficient in stabilising hydrophilic drug suspensions such as AZA due to its lower density of hydroxyl groups and limited hydrogen bonding ability [77]. The combined effect of electrostatic stabilisation of chitosan and steric hindrance by HPMC results in a strong electrosteric stabilisation mechanism that effectively prevents both aggregation and crystal growth [43]. This synergistic approach ensures the formation of stable AZA nanosuspensions with improved physical stability and bioavailability.

### 4.4. Implications for Dermal Drug Delivery

These findings highlight the importance of selecting complementary stabilisers to optimise the formulation of AZA-NS. The combination of HPMC and chitosan provided a good balance between steric and electrostatic stabilisation and ensured both short-term physical stability and processability. The improved wettability and reduced crystallinity of AZA10-H_3.4 and AZA10-HC suggest that these formulations have potential to improve bioavailability of AZA, which is critical for effective delivery to the skin. In addition, the shear-thinning behaviour of these nanosuspensions is suitable for topical application, where easy spreadability and stability at rest are important.

The dual stabilisation approach with HPMC and chitosan successfully improved the stability, processability and wettability of AZA-NS, making it a promising formulation strategy for dermal drug delivery. Overall, these results provide a strong foundation for further investigation of AZA-NS formulations in in vivo studies to evaluate their therapeutic performance and clinical potential in the treatment of skin disorders.

## 5. Conclusions

This study shows that the combination of HPMC and chitosan is an effective dual stabilisation system for AZA nanosuspensions (AZA-NS), which improves colloidal stability, wettability and processability. The results confirm that the AZA concentration, pH and composition of the stabiliser significantly influence the key properties of the nanosuspension, including the viscosity, rheological behaviour and retention of crystallinity, which are critical parameters for dermal drug delivery.

All formulations exhibited shear-thinning behaviour, with HPMC-based systems showing the highest viscosity and structural integrity. In contrast, chitosan alone resulted in lower network stability, while the HPMC–chitosan combination improved flowability, making these systems more suitable for dermal application and patient-friendly use.

XRD confirmed the retention of the crystalline structure, with a pH-dependent reduction observed for AZA10-H_3.4. Wettability tests showed better surface coverage with HPMC and chitosan, while P407 performed poorly. FTIR confirmed physical interactions between AZA and stabilisers, and DSC showed lattice disorder in chitosan-based systems.

Overall, the results provide a solid foundation for the development of stable, scalable nanosuspension-based formulations for the skin, particularly for follicular delivery for diseases such as acne and rosacea. Future work will apply the Design of Experiments (DoE) to optimise formulation and processing for improved clinical translation.

## Figures and Tables

**Table 1:** Composition and milling parameters of AZA-NS samples.

Sample ^1^	AZA (% *w*/*w*)	HPMC (% *w*/*w*)	Chitosan (% *w*/*w*)	P407 (% *w*/*w*)	pH	Milling Time (min)
AZA10-H_3.4	10	1.5	-	-	3.4	45 ^2^
AZA10-H	10	1.5	-	-	4.5	60
AZA20-H	20	1.5	-	-	4.5	60
AZA5-C0.1	5	-	0.1	-	4.5	60
AZA10-HC	10	1.5	0.2	-	4.5	60
AZA20-HC	20	1.5	0.2	-	4.5	60
AZA5-P	5	-	-	1.25	3.3	60

^1^ Sample nomenclature: 5, 10 or 20—indicates the concentration of AZA in the formulation (5%, 10% or 20%); H—formulation containing HPMC; H_3.4—formulation containing HPMC with a pH of 3.4; C0.1—chitosan at a concentration of 0.1%; HC—formulation containing both HPMC and chitosan; P—formulation containing P407. ^2^ Recirculation stopped.

**Table 2:** Physicochemical properties and short-term stability of AZA-NS. Values represent mean ± standard deviation (*n* = 3).

Formulation	Z-Average (nm) Day 0	Z-Average (nm) Day 10	PDI Day 0	PDI Day 10	Zeta Potential (mV) Day 0	Zeta Potential (mV) Day 10	Crystal Growth ^1^
AZA10-H_3.4	539.4 ± 8.19	555.2 ± 8.05	0.229 ± 0.006	0.183 ± 0.054	−2.85 ± 0.22	−2.84 ± 0.32	No
AZA10-H	834.8 ± 62.5	1205 ± 74.57	0.284 ± 0.010	0.377 ± 0.021	−4.35 ± 0.30	7.81 ± 0.48	No
AZA20-H	541.2 ± 30.0	613.7 ± 6.89	0.227 ± 0.015	0.302 ± 0.047	−3.36 ± 0.18	−3.13 ± 0.16	No
AZA5-C0.1	554.2 ± 12.50	803.9 ± 8.95	0.323 ± 0.015	0.343 ± 0.013	39.1 ± 1.0	30.5 ± 0.78	Yes
AZA10-HC	541.7 ± 16.98	613.9 ± 6.35	0.233 ± 0.027	0.245 ± 0.010	12.0 ± 0.80	15.0 ± 0.20	No
AZA20-HC	326.8 ± 2.60	414.9 ± 3.97	0.208 ± 0.012	0.246 ± 0.017	18.0 ± 0.57	21.6 ± 0.15	Yes

^1^ Observed on the microscopic image; samples with identified crystals larger than 2 µm are labelled Yes for crystal growth (AZA5-C0.1—acicular crystals up to 10 µm in diameter that were sporadically present to a similar extent on day 0 and day 10; AZA20-HC—elongated, rectangular crystals up to 20 µm in diameter that were present in the entire sample only on day 10).

**Table 3:** Contact angles and immersion time of AZA in purified water and stabiliser solutions (*n* = 6, x ± SD). Different superscript letters indicate statistically significant differences (*p* < 0.05).

Material	Initial Contact Angle (°)	Equilibrium Contact Angle (°)	Difference (°)	Immersion Time
1.5% HPMC	65.5 ± 3.0 ^b^	51.7 ± 4.0 ^b^	13.8 ± 3.1 ^b^	Immediately
0.2% Chitosan	69.9 ± 5.3 ^b^	56.4 ± 3.7 ^a^	13.5 ± 4.0 ^b^	>1 h (not wettable)
1.5% HPMC + 0.2% Chitosan	77.8 ± 4.9 ^a^	45.8 ± 3.1 ^c^	32.0 ± 2.7 ^a^	Immediately
1.25% P407	59.2 ± 3.5 ^c^	48.2 ± 2.1 ^c^	11.1 ± 3.0 ^c^	Immediately
Water	59.9 ± 3.5 ^c^	51.0 ± 2.1 ^b^	9.0 ± 2.5 ^c^	>1 h (not wettable)

Legend: Superscript letters (a, b, c) indicate statistically significant differences between the groups (*p* < 0.05). Different letters within a column indicate significant differences between samples. Samples with the same superscript letter within a column do not differ significantly from each other.

**Table 4:** Thermal properties of AZA and nanosuspension formulations determined by DSC.

Sample	$\omega_{polymer}$	MP (°C)	Onset Temp. (°C)	Enthalpy (J/g)	RDC (%)	MP Depression (°C)
AZA (Pure)	-	108.55	107.01	204.09	100.00	-
AZA-H PM	0.13	107.82	105.93	180.80	101.83	−1.08
AZA10-H_3.4	0.13	106.41	103.96	184.86	104.11	−3.05
AZA-C PM	0.13	108.31	106.80	184.10	103.68	−0.21
AZA5-C0.1	0.02	102.66	98.16	191.47	95.73	−8.85
AZA-HC PM	0.23	107.06	105.98	152.64	97.13	−1.03
AZA10-HC	0.15	104.58	100.23	162.51	93.68	−6.78
AZA-P407 PM	0.20	105.63	104.94	123.97	75.93	−2.07
AZA5-P	0.20	106.37	103.06	139.99	85.74	−3.95

Legend: MP—melting point; RDC—relative degree of crystallinity; MP depression—difference in onset melting temperature compared to pure AZA; PM—physical mixture; $\omega_{polymer}$—fraction of polymer in the mixture.

## Data Availability

Data are included in the article.

## Supplementary Materials

The following supporting information can be downloaded at: https://www.mdpi.com/article/10.3390/pharmaceutics17040439/s1, Table S1: Z-average, PDI, appearance, pH and resuspendability for 5% AZA-NS with different stabilisers, together with solubility, immersion time and contact angle. Figure S1. Fourier transform infrared spectroscopy (FTIR) spectra of azelaic acid (AZA) nanosuspensions (AZA-NS) and AZA-P407 suspension (AZA5-P) and corresponding physical mixtures (PM) prepared in 1:1 ratio. Figure S2. X-ray diffraction (XRD) patterns of pure AZA, HPMC, chitosan, P407 and corresponding physical mixtures (PM) of AZA and excipients prepared in 1:1 ratio. Figure S3. Differential scanning calorimetry (DSC) thermograms of AZA, AZA-NS formulations and AZA5-P suspension and corresponding AZA/stabiliser physical mixtures (PM) at ratio reflecting formulation (a) and 1:1 ratio (b). Figure S4. Differential scanning calorimetry (DSC) thermograms of AZA, HPMC, chitosan, P407, AZA-NS formulations and AZA5-P suspension.

## Abbreviations

The following abbreviations are used in this manuscript:

API	Active pharmaceutical ingredient
AZA	Azelaic acid
HPMC	Hydroxypropyl methylcellulose
AZA-NS	Azelaic acid nanosuspensions
WMM	Wet media milling
API	Active pharmaceutical ingredient
DLS	Dynamic laser scattering
ATR-FTIR	Attenuated total reflection Fourier transform infrared spectroscopy
DSC	Differential scanning calorimetry
RDC	Relative degree of crystallinity
XRD	X-ray diffraction
AFM	Atomic force microscopy
ECA	Equilibrium contact angle
PDI	Polydispersity index
P407	Poloxamer 407
LVR	Linear viscoelastic region
