# Supplementary material for: Characterisation and Stabilisation Mechanisms of Azelaic Acid Nanosuspensions: Insights from a Dual Stabiliser System
Source: Pharmaceutics. 2025 Mar 29;17(4):439. doi: 10.3390/pharmaceutics17040439 (PMC12030390; doi:10.3390/pharmaceutics17040439)
Supplement: Supplementary file 1 [file pharmaceutics-17-00439-s001.zip › pharmaceutics-3528665-supplementary.pdf]

Table S1. Z-average, PDI, appearance, pH and resuspendability for 5% AZA-NS with different stabilisers, together with solubility, immersion time and contact angle[a].

Stabiliser	% (w/w) Stabiliser	Z-average (nm) ± SD (n=3)	PDI ± SD (n=3)	AZA solubility (mg/mL)± SD (n=3)[b c]	Immersion time (s)	Contact angle (º) ± SD (n=6)			Appearance[d]	pH	Re-Suspendability (after 3 months at RT)
						Initial	Equilibrium	Angle Decrease			
**Polysorbate 20**	0.5	3089±203.5	0.347±0.026	3.59±0.27	Immediately	60.2± 3.7	45.0± 2.8	15.2±3.9	White liquid	3.5	No
**Polysorbate 80**	1.2	*766±14.3*	0.210±0.028	3.98±0.25	Immediately	58.0±2.8	44.6±3.0	13.4±2.6	White liquid	3.5	Yes
**Alkylpolyglucoside (*Plantacare® 188UP*)**	0.5	2324±157	0.712±0.142	3.21±0.13	Immediately	60.7±4.7	37.6±4.6	23.1±6.4	White liquid	3.4	No
**Vitamin E TPGS**	1.0	2084±186	0.566±0.129	2.32±0.02	Partially[c]	68.2±1.8	58.7±2.0	9.5±2.4	White liquid	3.5	No
**Tyloxapol**	0.5	1347±43.4	0.288±0.034	2.01±0.04	30 s	66.7±2.0	47.6±3.7	19.1±2.9	Foamy suspension	3.5	Yes
**Poloxamer 407**	1.25	1100±11.7	0.227±0.009	3.52±0.11	Immediately	59.2±3.5	48.2±2.1	11.1±3.0	White liquid	3.3	No
**Hydroxypropyl Cellulose (*Klucel®EXF*)**	2.5	1393±40.7	0.369±0.075	1.98±0.1	8 s	62.9±2.9	50.5±2.9	12.5±2.1	White liquid	3.5	No
**HPMC (*Methocel® LV E5*)**	1.5	*918.3±98.5*	0.237±0.014	2.61±0.42	Immediately	65.5±3.0	51.7±4.0	13.8±3.1	White liquid	3.5	Yes
**Carboxymethyl Cellulose (CMC) Sodium (*Blanose® 7L2P*)**	1.25	1687±55.0	0.286±0.038	3.84±0.48	Not wettable	74.5±2.8	50.6±6.0	23.9±5.4	Aggregates visible	4.7	Yes
**Sodium Docusate**	0.5	2456±204	0.422±0.088	3.48±0.12	Immediately	37.3±6.1	21.2±4.3	16.1±5.2	White liquid	3.5	Yes
**Chitosan**	0.1	*554.2±12.5*	0.323±0.015	2.49±0.08	Not wettable	69.9±5.3	56.4±3.7	13.5±4.0	Foam on top[e]	4.5	Yes
**Benzalkonium Chloride**	0.1	1112±32.3	0.250±0.012	2.00±0.01	15 s	66.9±5.6	48.2±2.8	18.72±4.95	White liquid	3.5	Yes
**Polyethylenimine**	0.5	1182±25.1	0.313±0.032	6.07±0.23	Partially[c]	68.5±4.8	57.6±5.2	10.9±4.2	Foam on top[e]	4.4	Yes
**L-arginine**	1.0	3632±420	0.524±0.15	6.64±0.63	Not wettable	78.6±3.1	53.4±3.8	25.2±6.6	Foam on top[e]	4.9	No

Table S1. Z-average, PDI, appearance, pH and resuspendability for 5% AZA-NS with different stabilisers, together with solubility, immersion time and contact angle[a] **(cont.)**

Stabiliser	% (w/w) Stabiliser	Z-average (nm) ± SD (n=3)	PDI ± SD (n=3)	AZA solubility (mg/mL)± SD (n=3)[b c]	Immersion time (s)	Contact angle (º) ± SD (n=6)			Appearance[d]	pH	Re-Suspendability (after 3 months at RT)
						Initial	Equilibrium	Angle Decrease			
**Lecithin**	0.25%	1740±82.4	0.325±0.012	1.93	Not wettable	80.6±1.5	70.8±3.0	9.8±2.1	Foam on top	3.5	Yes
**Soluplus**	0.5%	2715±156.4	0.267±0.047	2.83	Partially	72.0±2.5	55.2±4.1	16.8±2.7	White liquid	3.5	No

[a]All formulations contained 5% AZA, pH was not adjusted, milling parameters were as described in the article; target milling time was 120 minutes but varied as some stabilisers clogged and milling time was shorter (chitosan, polyethylamine, hydroxypropylcellulose - 60 minutes, HPMC; lecithin, Soluplus – 45 minutes, carboxymethylcellulose – 90 minutes)

[b]Solubility was tested in 0.3 % aqueous chitosan solution and in 3.0 % HPMC solution.

[c]Partially wettable - a small amount of the powder remained floating on the liquid, while most of it sank immediately.

[d]Chitosan, CMC sodium, benzalkonium chloride, lecithin, L-arginine and PEI – needle-shaped crystals visible on the microscopic image.

[e]Phase separation of the suspension occurred - foamy upper layer, lower clear layer.

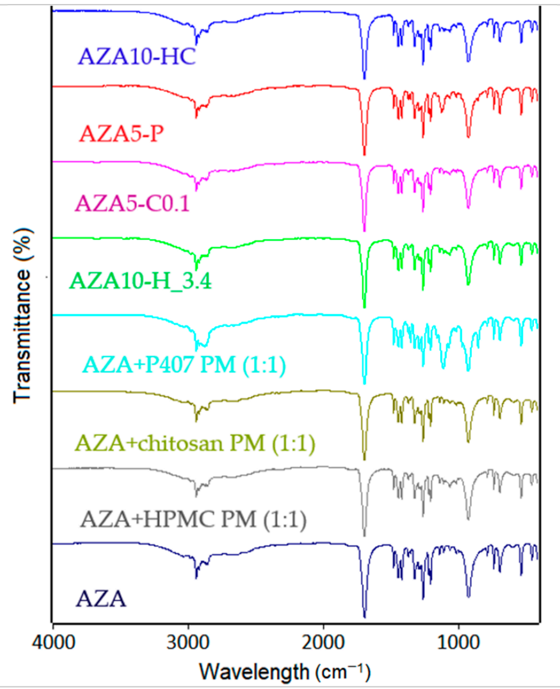

Figure S1. Fourier transform infrared spectroscopy (FTIR) spectra of azelaic acid (AZA) nanosuspensions (AZA-NS) and AZA-P407 suspension (AZA5-P) and corresponding physical mixtures (PM) prepared in 1:1 ratio.

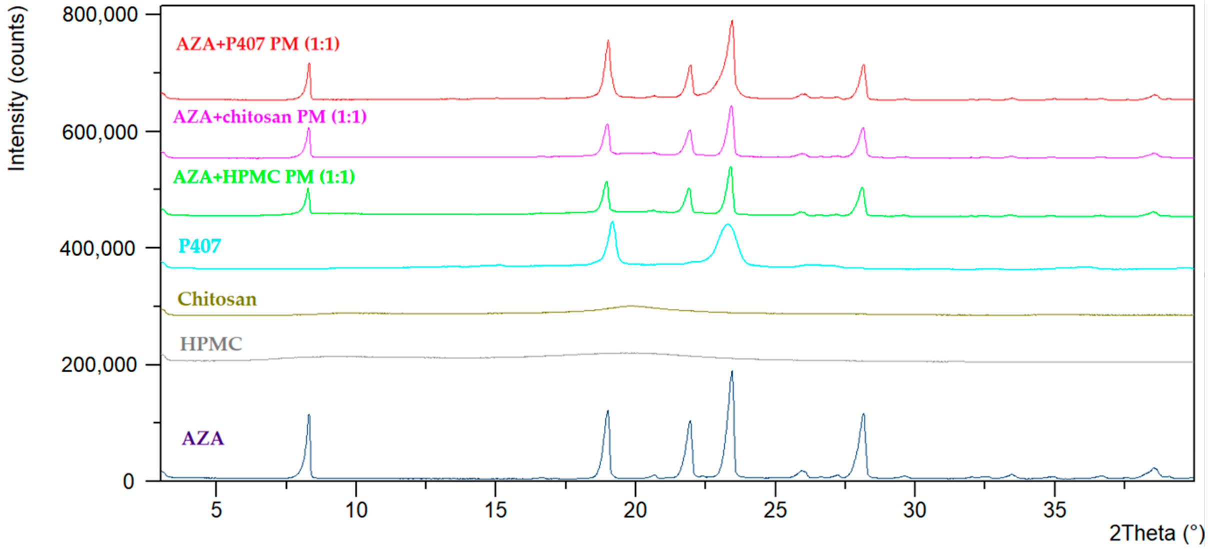

Figure S2. X-ray diffraction (XRD) patterns of pure AZA, HPMC, chitosan, P407 and corresponding physical mixtures (PM) of AZA and excipients prepared in 1:1 ratio.

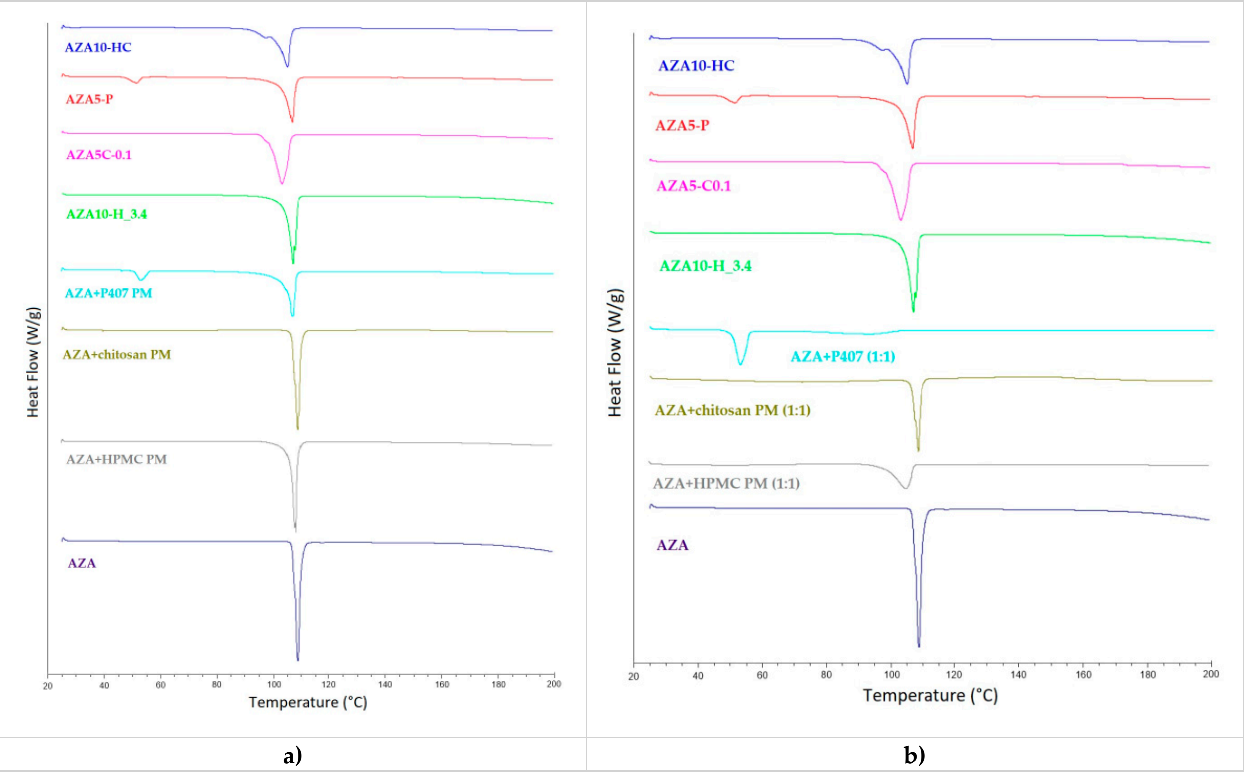

Figure S3. Differential scanning calorimetry (DSC) thermograms of AZA, AZA-NS formulations and AZA5-P suspension and corresponding AZA/stabiliser physical mixtures (PM) at ratio reflecting formulation (a) and 1:1 ratio (b).

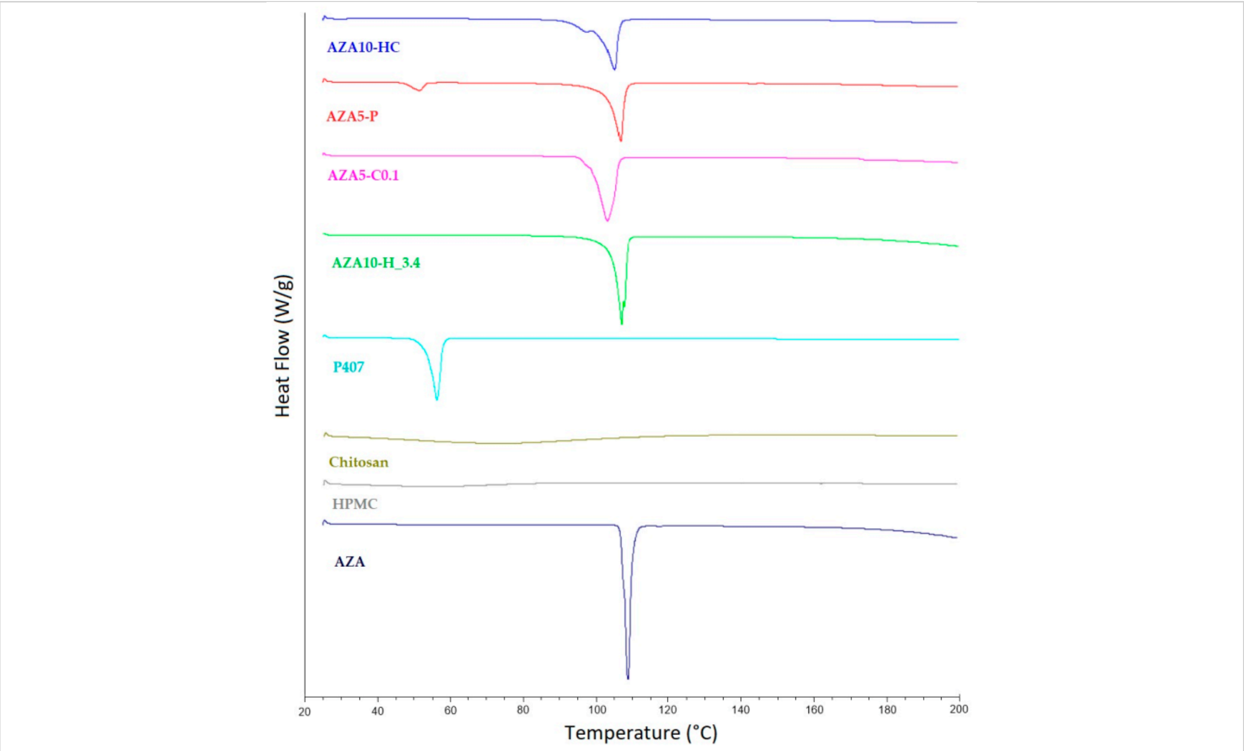

Figure S4. Differential scanning calorimetry (DSC) thermograms of AZA, HPMC, chitosan, P407, AZA-NS formulations and AZA5-P suspension.
